# Blood Pressure and Global Risk Assessment in a Swedish Population

**DOI:** 10.1155/2012/835812

**Published:** 2012-09-06

**Authors:** Jenny Eckner, Charlotte A. Larsson, Lennart Råstam, Ulf Lindblad

**Affiliations:** ^1^Department of Primary Health Care, Institute of Medicine, Sahlgrenska Academy, University of Gothenburg, Arvid Wallgrens Backe 4, Hus 7, P.O. Box 454, 405 30 Gothenburg, Sweden; ^2^Department of Clinical Sciences/Community Medicine, Lund University, Clinical Research Centre, SUS Malmö, 205 02 Malmö, Sweden

## Abstract

This study investigated the association between SCORE and the 2007 ESH-ESC blood pressure categories and explored achievements of blood pressure goals considering global risk. In 2001–2005, a random sample of inhabitants aged 30–74 years in southwestern Sweden was invited to a survey of cardiovascular risk factors. The study enrolled 2816 participants (participation rate 76%). Blood pressure was categorized according to the 2007 ESH-ESC guidelines. Global risk of 10-year CVD death was estimated using the Swedish SCORE chart also accounting for additional risk from diabetes (SCORE-DM). SCORE-DM increased in both sexes from optimal blood pressure to manifest hypertension but did not differ between the normal blood pressure categories. However, SCORE-DM became significantly higher among those with temporarily high blood pressure (men 3.3 SD (1.7), women 1.1 (1.8)) and hypertension (3.6 (2.0), 2.0 (2.0)), compared to optimal blood pressure (1.6 (2.9), 0.6 (1.9)). In the presence of both hypertension and diabetes, high-risk subjects dominated (men 76%, women 61%), and correspondingly a major proportion of patients with known hypertension were at high risk at a blood pressure ≥160/100 mm Hg. These findings have strong implications on blood pressure evaluation in clinical practice and support the use of SCORE to evaluate global risk.

## 1. Introduction

Population studies in Sweden and many other countries show that hypertension is a common condition [1], which seriously affects future health and quality of life [1]. It is also evident that other diseases and cardiovascular risk factors interact with high blood pressure in determining the individual global risk [2]. Those with both hypertension and diabetes have been identified to be at a special high risk of complications [3, 4]. To help in correctly selecting the individuals in the highest need of treatment, special risk grading tools have been developed considering other cardiovascular risk factors to calculate a global risk score (SCORE) [2, 5], and also accounting for the risk added by diabetes [6]. When the 10-year mortality risk is at least 5%, pharmacological treatment is recommended [2]. 

Recent studies in Skaraborg, Sweden, show a prevalence of manifest hypertension at 20% among both men and women aged 30–75 [7]. Again only one half of those with manifest hypertension fulfilled recommended treatment goals [7]. However, neither the Skaraborg Study, nor other population based studies accounted for the global risk when evaluating blood pressure control in hypertension [8, 9].

The aim was to study the association between SCORE and blood pressure levels according to current European expert guidelines in this Swedish population [6]. In a second step, we explored achievements of blood pressure goals considering treatment recommended by high SCORE or not. 

## 2. Methods

### 2.1. The Skaraborg Project

#### 2.1.1. Study Population

The Vara-Skövde Cohort (VSC) was collected 2001–2005 as a random sample of subjects aged 30–74 years residing in these two small municipalities in southwestern Sweden. Of a total 2816 subjects 1400 were men and 1416 women and the participation rate 76% as described in detail before [10]. 

### 2.2. Procedures

#### 2.2.1. Measurements

The participants provided detailed information on medical history and ongoing medication and filled in a validated questionnaire regarding life styles. A standard blood pressure (right A. Brachialis) to the nearest 2 mm Hg was measured twice in a supine position, with one minute in between. The arm was placed in heart level by the support of a special pillow, and the cuff was automatically adjusted to the circumferences of the upper arm using a special device [11]. The mean of the two blood pressure readings was used for categorisation and for analyses. Body height (nearest cm) and body weight (nearest 0.1 kg) were measured in light clothing and without shoes with a calibrated scale. Waist circumference was measured to the closest cm between the lowest rib margin and iliac crest and hip circumference correspondingly at the largest circumference between waist and thighs. Body mass index (BMI kg m^−2^) was calculated as body weight (kg) divided by body height^2^ (m^2^). Fasting venous blood samples were drawn in the morning after an overnight 10 h fast, and a standard oral glucose tolerance test (OGTT) was conducted according to WHO [12]. 

#### 2.2.2. Blood Pressure Categories

Untreated subjects with a blood pressure of at least at 140/90 mm Hg were seen again at a second visit within 2 weeks, and if still ≥140 and/or 90 mm Hg, they were seen a third time again within 2 weeks. Those with a high blood pressure at the first study visit who had a normal blood pressure (<140/<90 mm Hg, both systolic and diastolic) at the second or third visit were considered normotensive. However, for this paper, they were considered to have an unstable blood pressure. Normal blood pressure was defined in accordance with the 2007 ESH-ESC Guidelines [6] and further divided into three categories: normal optimal (≤120/80 mm Hg), normal (≤130/85 mm Hg), and normal high (<140/90 mm Hg). If SBP and DBP belonged to different categories, the highest was chosen. 

### 2.3. Definitions

A diagnosis of hypertension was considered when three consecutive high readings with two-week intervals (≥140 systolic and/or ≥90 mm Hg diastolic) were registered [3], or when a known diagnosis set by a physician was documented. Manifest hypertension was further categorised as grade 1 (140–159 mm Hg systolic and/or 90–99 diastolic), grade 2 (160–179 systolic and/or 100–109 diastolic), or grade 3 hypertension (≥180 systolic and/or ≥110 diastolic) in accordance with current European guidelines. International guidelines have been used for the diagnosis of type 2 diabetes [13].

### 2.4. Risk Score Algorithm

The score chart of Sweden was used to estimate the 10-year risk of cardiovascular death, accounting for risks based on sex, age, systolic blood pressure, total serum cholesterol, and on current smoking [2, 5]. Based on individual values in these variables, all participants were placed in the corresponding score cell thus to be assigned a risk estimation according to SCORE. The corresponding risk accounting for the risk added by the presence of diabetes was calculated by the multiplication by 2 in men and by 4 in women [5, 6]. As suggested in SCORE [2, 5], the global risk was considered as high if the 10-year risk of cardiovascular death ≥5 percent (SCORE-HIGH), and correspondingly low if <5 percent. For SCORE-DM, score with diabetes included, the same procedure was done, and considered high if the 10-year risk of cardiovascular death ≥5 percent (SCORE-DM-HIGH). 10 percent of the participants were randomly chosen to have their score manually calculated from the chart. The 10-year mortality risk was accordingly considered high or low and was in all cases found to be the same as when based on the algorithm in all participants. 

### 2.5. Statistical Analysis

SPSS Base System for Windows 19.0 was used for data analyses. All proportions of the study population were age-standardized by five-year age groups using the whole Skövde-Vara population 30–75 years as standard, while means were adjusted for differences in age using general linear model (GLM). GLM was used to compare means between groups in continuous variables, and results were given as differences with 95% confidence intervals (95% CI). Logistic regression was used to estimate associations between categorical variables, and results were presented as odds ratio (OR) with 95% confidence intervals (CI). Confounding was accounted for by multivariate analyses and by stratification. All tests were 2 sided, and statistical significance was assumed if *P* < 0.05.

## 3. Results 

**Figure 1:**
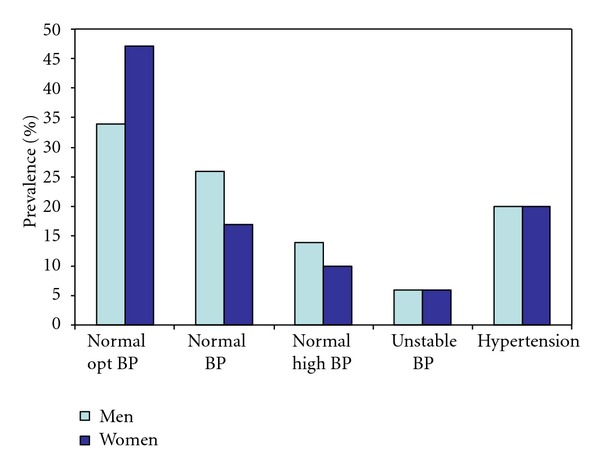
The figure illustrates the distribution (%) of blood pressure (BP) categories according to the 2007 ESH-ESC guidelines in men and women, respectively. The 2007 ESH-ESC blood pressure categories; normal optimal BP <120/80 mm Hg, normal BP <130/85 mm Hg, normal high BP <140/90 mm Hg. Hypertension was defined as known documented diagnosis for high blood pressure, or by three consecutive BP reading ≥140/90 mm Hg (systolic and/or diastolic). When the BP exceeded these limits only once or twice, the BP was categorised as unstable.

The distribution of blood pressure categories according to the 2007 European guidelines is shown in Figure 1. The overall proportion of normal blood pressure was 74%, of unstable blood pressure 6%, and of hypertension 20% in both men and women. The normal optimal blood pressure was the most common category in both men and women.  Table 1 shows that 13% of the men and 1% of the women were categorized as high risk (10-year risk of cardiovascular death ≥5%), when also diabetes was considered the corresponding proportions were 14% and 4%, respectively. 

Table 2 shows the study characteristics by blood pressure categories. The mean risk scores did not differ between the normal blood pressure categories. However, in both sexes, SCORE became significantly higher among those with unstable blood pressure and manifest hypertension, respectively, than in those with optimal blood pressure. The proportion of subjects defined as high risk increased from normal blood pressure (2%) to hypertension (46%) in men, and correspondingly from 0% to 7% in women. When diabetes risk was accounted for, the proportions increased from 3% to 50% in men and from 0% to 19% in women. In subjects with known hypertension, these proportions were considerably higher when treatment goals were not met; 60% in men and 43% in women when blood pressure was ≥160/100 mm Hg (grade 2 hypertension), and 57% and 100%, respectively, when ≥180/110 mm Hg (grade 3 hypertension).

**Figure 2:**
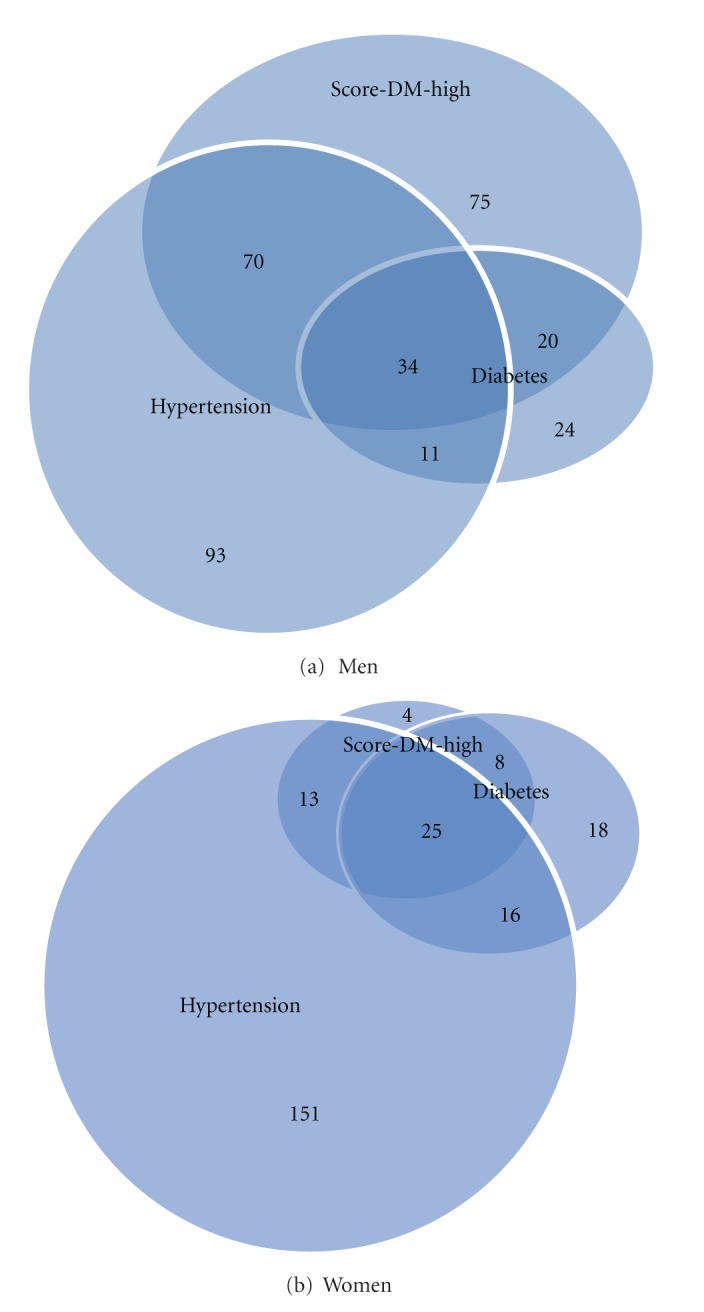
Venn diagrams in men (a) and women (b), showing the overlap between categories of hypertension, diabetes, and high risk score, respectively, accounting for diabetes.

Characteristics of high and low risk subjects, based on SCORE-DM-HIGH, are shown in Figure 2. In the presence of both hypertension and diabetes, high-risk subjects dominate, 76% in men and 61% in women.  Table 3 shows accordingly levels of risk factor variables included in SCORE-DM stratified by SCORE-DM being high or not. In low risk subjects, even in the presence of both diabetes and hypertension, age and concentrations of lipids were low. Men and women with high score without any diagnosis of hypertension or diabetes were older and had higher concentrations of lipids and were often smoking. Finally all analyses were repeated among subjects aged 40–65 years, and the results were consistent with those found in the whole study population (30–75 years). 

## 4. Discussion

Traditional CVD risk factors increased in both men and women the higher the blood pressure category. The accumulation of risk factors among subjects with manifest hypertension and unstable blood pressure was confirmed by the global risk score that significantly separated hypertension and unstable blood pressure from all the normal blood pressure categories according the 2007 European guidelines. This pattern was even more pronounced when risk score also accounted for diabetes. Most subjects with manifest hypertension had an estimated 10-year CVD mortality risk <5% and should thus not by routine be prescribed pharmacological treatment.

Blood pressure levels that are recommended for therapy by expert guidelines are according to this study accurately decided based on SCORE. However, global risk estimation by SCORE did not identify all subjects with a diagnosis of hypertension and/or diabetes as being at a high risk. Many study subjects with hypertension, especially women, did not reach a SCORE that indicates the need of drug treatment. Instead this study showed that a substantial proportion of men having high SCORE had neither hypertension nor diabetes. The high SCORE risk was mainly attributed to old age if hypertension or diabetes were not present (Table 3). When both hypertension and diabetes was present, high-risk SCORE predominated, which confirms the hazard of having both. 

A major strength of this study was the high participation rate, which gives the results a high trustworthiness. It is still likely that individuals with chronic diseases or health problems prior to the study would be more reluctant to participate than healthy people, as often seen in other surveys [14]. Nevertheless, this is not likely to have had a considerable impact on the observed prevalence. Another strength of the study was the accurate blood pressure measurements and the strict diagnostic procedures, thus limiting the risk of overestimating unaware hypertension due to randomly high blood pressure levels [15].

Originally SCORE did not consider risk from diabetes, but a modified algorithm has been proposed by multiplying the score value by two in men and by four in women, which we accordingly did [5, 6]. This also gave us the opportunity to compare the different score models in relation to the blood pressure categories in the 2007 ESH-ESC guidelines [6]. However, as expected in a healthy population, they were very similar. Originally SCORE did not include risk assessments for systolic blood pressure above 180 mm Hg or a total for serum cholesterol above 8 mmol/L. At these levels, the risks are considered so high that treatment is still indicated independently of the global risk assessment [2]. In our study population, only 23 subjects (0.8%) had systolic blood pressure ≥180 mm Hg, and only 34 subjects (1.2%) had a total serum cholesterol ≥8 mmol/L. Originally score was constructed for the age range between 40 and 65 years. As age is such a strong risk factor almost all men older than 65 years have a mortality risk of 5 percent or more regardless of their cholesterol level, blood pressure, or smoking habits. For cardiovascular risk assessment to achieve increased utilising in primary care, we decided to include all subjects in the original study population. Still, including subject ≥65 years of age would rather tend to increase the proportion with a high risk that would accordingly be recommended pharmacological treatment. Thus, the small proportion with a low 10-year mortality risk was probably not an underestimation.

We have found that a large proportion of patients with hypertension had a low risk according to SCORE. This contradicts previous reports suggesting that SCORE overestimates cardiovascular risk [16]. This in turn may partially advocate our recent findings that only one-third of all subjects with treated hypertension achieve recommended blood pressure goals [7]. We may have to accept that some subjects, especially women, are considered adequately cared for despite not having a blood pressure ≤140/90 mm Hg. However, in patients with grade 2 or grade 3 hypertension the estimated mortality risk was considerably higher emphasizing the need of more attention for these patients. 

The present findings imply that cardiovascular risk estimation using SCORE is parallel with risk increase according to 2007 ESH-ESC blood pressure categories and general expert treatment guidelines. Our findings may have strong implications on blood pressure evaluation in clinical practice and emphasize the need of nonpharmacological interventions among subjects with high normal blood pressure and low risk hypertension [17–19]. These questions should be further investigated in longitudinal population-based studies. 

## Figures and Tables

**Table 1:** Study characteristics of men and women participating in the Vara-Skövde population survey 2001–2005.

	Men		Women	
	n = 1400		n = 1416	
	m	(SD)	m	(SD)
Age (years)	47.8	(11.8)	47.8	(11.7)
Systolic blood pressure (mm Hg)	124	(14.2)	119	(14.2)
Diastolic blood pressure (mm Hg)	72	(9.5)	69	(9.6)
Serum cholesterol (mmol L^−1^)	5.3	(1.0)	5.2	(1.0)
SCORE^a^	1.6	(1.6)	0.5	(1.6)
SCORE-DM^b^	1.9	(2.5)	0.8	(2.5)

	*n*	(%)	*n*	(%)

Daily smoking	216	(15)	289	(20)
Known diabetes^c^	51	(5)	42	(4)
SCORE-HIGH^d^	179	(13)	19	(1)
SCORE-DM-HIGH^e^	200	(14)	52	(4)

^a^ SCORE: risk score according to the original model, with missing values for 6 men and 4 women.

^b^SCORE-DM: risk score considering diabetes, with missing values for 7 men and 6 women.

^c^Self-reported doctors diagnosis of diabetes (type 1 or type 2).

^d^SCORE-HIGH: 10-year risk of cardiovascular death ≥5 percent, with missing values for 6 men and 4 women.

^e^SCORE-DM-HIGH: 10-year risk of cardiovascular death ≥5 percent considering diabetes, with missing values for 5 men and 3 women.

**Table 2:** Comparison of common cardiovascular disease risk factors between categories of hypertension in men and women using aware controlled hypertension as reference. The Vara-Skövde Cohort 2001–2005 within the Skaraborg Project.

	Normal blood pressure			Unstable blood pressure	Hypertension
Men	Optimal (*n* = 557)	Normal (*n* = 385)	High (*n* = 179)	(*n* = 70)	(*n* = 209)

Age					
Mean (SD)	42.8 (8.5)	45.0 (10.0)	51.9 (12.3)	55.4 (11.1)	60.5 (10.5)
Diff (CI)		2.2 (1.0; 3.5)	9.2 (7.5; 10.9)	12.7 (10.2; 15.1)	17.7 (16.1; 19.3)
Systolic BP (mm Hg)					
Mean (SD)	110.9 (8.9)	123.4 (8.5)	132.9 (8.5)	144.2 (8.6)	144.9 (9.4)
Diff (CI)		12.5 (11.4; 13.6)	22.0 (20.5; 23.4)	33.2 (31.1; 35.4)	34.0 (32.4; 35.5)
Diastolic BP (mm Hg)					
Mean (SD)	66.0 (8.3)	71.4 (8.0)	76.9 (8.0)	83.7 (8.0)	81.8 (8.8)
Diff (CI)		5.8 (4.8; 6.9)	11.4 (10.0; 12.8)	18.2 (16.1; 20.2)	16.3 (14.8; 17.8)
Total chol (mmol/L)					
Mean (SD)	5.3 (1.1)	5.4 (1.0)	5.4 (1.0)	5.7 (1.0)	5.2 (1.1)
Diff (CI)		0.1 (−0.1; 0.2)	−0.2 (0.0; 0.3)	0.4 (0.1; 0.6)	−0.1 (−0.3; 0.1)
SCORE					
Mean (SD)	1.4 (1.9)	1.2 (1.9)	1.2 (1.9)	2.8 (1.9)	2.7 (2.1)
Diff (CI)		−0.1 (−0.3; 0.1)	−0.1 (−0.4; 0.2)	1.5 (1.0; 2.0)	1.4 (1.0; 1.7)
SCORE-DM					
Mean (SD)	1.6 (2.9)	1.4 (2.8)	1.2 (2.8)	3.3 (1.7)	3.6 (2.0)
Diff (CI)		−0.2 (−0.5; 0.2)	−0.3 (−0.8; 0.2)	1.7 (1.0; 2.4)	2.0 (1.5; 2.6)
Smoking					
*n* (%)	87 (16)	59 (14)	30 (17)	15 (22)	25 (12)
OR (CI)		1.0 (0.7; 1.4)	1.1 (0.7; 1.7)	1.5 (0.8; 2.9)	0.7 (0.4; 1.3)
Diabetes					
*n* (%)	15 (3)	12 (3)	10 (6)	7 (10)	45 (22)
OR (CI)		1.0 (0.5; 2.2)	1.2 (0.5; 2.9)	2.0 (0.7; 5.3)	3.9 (1.9; 8.0)
CVD					
*n* (%)	7 (1)	5 (1)	11 (6)	1 (1)	36 (17)
OR (CI)		0.7 (0.2; 2.2)	1.4 (0.5; 4.1)	0.2 (0.0; 2.1)	2.6 (1.0; 6.5)
SCORE-HIGH					
*n* (%)	12 (2)	19 (5)	28 (16)	25 (37)	95 (46)
OR (CI)		1.3 (0.5; 3.9)	1.3 (0.5; 3.7)	10.2 (3.2; 33.2)	4.9 (1.9; 12.2)
SCORE-HIGH-DM					
*n* (%)	15 (3)	20 (5)	33 (18)	28 (41)	104 (50)
OR (CI)		1.0 (0.3; 2.6)	1.3 (0.5; 3.5)	10.1 (3.3; 30.5)	4.7 (2.0; 11.2)

Women	Optimal (*n* = 805)	Normal (*n* = 230)	High (*n* = 115)	(*n* = 60)	(*n* = 206)

Age					
Mean (SD)	42.2 (7.9)	48.7 (10.7)	55.4 (11.9)	58.2 (9.5)	61.1 (10.3)
Diff (CI)		6.5 (5.1; 7.8)	13.2 (11.4; 15.0)	16.0 (13.6; 18.4)	18.9 (17.5; 20.3)
Systolic BP (mm Hg)					
Mean (SD)	108.1 (9.6)	122.8 (8.7)	132.6 (8.9)	143.9 (8.9)	145.4 (9.9)
Diff (CI)		14.6 (13.3; 15.9)	24.5 (22.7; 26.3)	35.8 (33.4; 38.2)	37.2 (35.6; 38.9)
Diastolic BP (mm Hg)					
Mean (SD)	63.1 (8.6)	71.9 (7.8)	75.0 (8.0)	81.1 (8.0)	79.3 (8.9)
Diff (CI)		8.9 (7.7; 10.1)	12.0 (10.3; 13.6)	18.0 (15.8; 20.2)	16.2 (14.8; 17.7)
Total chol (mmol/L)					
Mean (SD)	5.2 (1.1)	5.3 (1.0)	5.4 (1.0)	5.5 (1.0)	5.1 (1.1)
Diff (CI)		0.2 (0.0; 0.3)	0.2 (0.0; 0.4)	0.3 (0.0; 0.6)	0.0 (−0.2; 0.1)
SCORE					
Mean (SD)	0.4 (0.8)	0.3 (0.7)	0.4 (0.8)	0.9 (0.8)	1.1 (0.8)
Diff (CI)		−0.1 (−0.2; 0.0)	−0.1 (−0.2; 0.1)	0.4 (0.2; 0.6)	0.6 (0.5; 0.8)
SCORE-DM					
Mean (SD)	0.6 (1.9)	0.3 (1.7)	0.4 (1.8)	1.1 (1.8)	2.0 (2.0)
Diff (CI)		−0.3 (−0.6; 0.0)	−0.2 (−0.5; 0.2)	0.5 (0.0; 0.9)	1.3 (1.0; 1.7)
Smoking					
*n* (%)	162 (20)	50 (22)	26 (23)	12 (20)	39 (19)
OR (CI)		1.2 (0.8; 1.7)	1.3 (0.8; 2.2)	1.2 (0.6; 2.3)	1.1 (0.7; 1.8)
Diabetes					
*n* (%)	11 (1)	5 (2)	6 (5)	5 (8)	42 (20)
OR (CI)		1.2 (0.4; 3.5)	2.2 (0.7; 6.5)	3.3 (1.0; 10.6)	8.2 (3.5; 19.0)
CVD					
*n* (%)	6 (1)	6 (3)	2 (2)	1 (2)	12 (6)
OR (CI)		1.1 (0.3; 3.7)	0.3 (0.0; 1.5)	0.3 (0.0; 2.4)	0.6 (0.2; 2.0)
SCORE-HIGH					
*n* (%)	1 (0)	0 (0)	2 (2)	2 (3)	14 (7)
OR (CI)		0.0 (0.0; —)	1.4 (0.4; 8.6)	3.0 (0.8; 16.9)	3.9 (1.8; 24.3)
SCORE-HIGH-DM					
*n* (%)	3 (0)	0 (0)	5 (4)	5 (8)	39 (19)
OR (CI)		0.0 (0.0; —)	1.8 (0.4; 8.6)	3.6 (0.8; 16.9)	6.6 (1.8; 24.3)

**Table 3:** Global risk according to SCORE characterized by SCORE variables in men and women of the Vara-Skövde Cohort in the Skaraborg Project 2001–2005.

	Mort ≥ 5%	DM	HT	Age	(SD)	SBP	(SD)	Chol	(SD)	Smoke	(%)
Men (*n*)											
1067	−	−	−	43.7	(8.5)	120.0	(13.7)	5.4	(1.1)	157	(15)
24	−	+	−	44.4	(8.6)	125.4	(13.8)	5.1	(1.0)	7	(29)
93	−	−	+	53.2	(8.2)	139.4	(13.3)	5.3	(1.0)	6	(7)
11	−	+	+	47.8	(11.8)	132.8	(13.8)	4.7	(1.0)	0	(0)
75	+	−	−	68.2	(4.6)	118.0	(15.0)	5.8	(1.1)	23	(31)
20	+	+	−	66.7	(5.7)	119.8	(14.1)	5.0	(1.0)	4	(20)
70	+	−	+	68.3	(4.9)	141.7	(14.4)	5.2	(1.1)	11	(16)
34	+	+	+	68.2	(5.8)	134.1	(14.3)	5.1	(1.0)	8	(24)

Women (*n*)											
1176	−	−	−	45.2	(10.1)	115.9	(12.9)	5.2	(1.0)	240	(20)
18	−	+	−	47.8	(11.7)	121.0	(14.4)	5.2	(1.0)	3	(17)
151	−	−	+	59.7	(10.4)	136.4	(14.0)	5.2	(1.0)	28	(19)
16	−	+	+	57.1	(10.1)	134.2	(14.3)	4.5	(1.0)	1	(6)
4	+	−	−	66.6	(4.4)	120.8	(14.4)	6.8	(1.0)	4	(100)
8	+	+	−	69.0	(4.2)	111.4	(14.5)	4.8	(1.0)	3	(38)
13	+	−	+	68.4	(9.3)	155.2	(14.2)	5.4	(1.0)	3	(17)
25	+	+	+	68.6	(3.8)	131.4	(14.7)	4.8	(1.0)	4	(16)
